# Phenotypes of Sarcopenic Obesity: Exploring the Effects on Peri-Muscular Fat, the Obesity Paradox, Hormone-Related Responses and the Clinical Implications

**DOI:** 10.3390/geriatrics5010008

**Published:** 2020-02-14

**Authors:** Tariq A. Alalwan

**Affiliations:** Department of Biology, College of Science, University of Bahrain, P.O. Box 32038, Sakhir, Bahrain; talalwan@uob.edu.bh; Tel.: +973-1743-7426

**Keywords:** obesity, sarcopenia, visceral fat, subcutaneous fat, inflammation

## Abstract

Sarcopenic obesity combines the words sarcopenia and obesity. This definition of obesity should be better differentiated between visceral and subcutaneous fat phenotypes. For this reason, this review lays the foundation for defining the subcutaneous and the visceral fat into the context of sarcopenia. Thus, the review aims to explore the missing links on pathogenesis of visceral fat and its relationship on age: defining the peri-muscular fat as a new entity and the subcutaneous fat as a first factor that leads to the obesity paradox. Last but not least, this review underlines and motivates the mechanisms of the hormonal responses and anti-inflammatory adipokines responsible for the clinical implications of sarcopenic visceral obesity, describing factor by factor the multiple axis between the visceral fat-sarcopenia and all mortality outcomes linked to cancer, diabetes, cardiovascular diseases, cirrhosis, polycystic ovary, disability and postoperative complications.

## 1. Introduction

The definition of sarcopenic obesity combines the definitions of sarcopenia (low muscle mass and strength) and obesity (excess adiposity). This definition is meaningless, however, due to the lack of established guidelines related to the visceral and subcutaneous adipose tissue [1]. A recent position statement on obesity claims the need for public health messaging to focus on visceral and ectopic fat, in addition to excess bodyweight to better combat the growing obesity epidemic worldwide [2]. Body changes in the elderly involve the loss of lean body mass while fat mass may be preserved or even increased in two different directions (visceral or subcutaneous), thereby giving rise to the specific condition of sarcopenic obesity [3].

According to a recent study by Perna et al. [4], the sarcopenic visceral obesity phenotype seems to be associated with inflammation, increased risk of fractures, and a worse metabolic profile. The authors of the same study concluded that such risks were associated with an increase in visceral adipose tissue, while elderly patients with sarcopenic subcutaneous obesity seem to benefit from lower mortality rates in what is known as the “obesity paradox.”

The age-related loss of muscle mass and gain in fat appear to be linked to each other and, together with positive energy balance, contribute to the development of phenotypes of sarcopenic obesity [5].

Perna et al. [1], in their editorial letter, commented that sarcopenic obesity is an obsolete concept that defines people who simultaneously have an excess of body fat greater than median or >27% in men and 38% in women and loss of muscle mass and strength as determined by dual-energy X-ray absorptiometry (DXA) or bioelectrical impedance analysis (BIA). In the elderly, a decline in muscle mass and physical activity leads to a lower metabolism, which in turn leads to weight gain and increase in abdominal fat [5].

An alternative definition of sarcopenic obesity has been recently proposed, suggesting the combination of low muscle strength (as opposed to muscle mass) and abdominal obesity, thereby introducing the concept of dynapenic obesity [6]. Nevertheless, the lack of consensus with regards to the appropriate diagnostic tools and criteria represent major shortcomings in terms of defining the concept of sarcopenic obesity that is easily adopted by clinical guidelines [7].

For all these reasons, it is imperative to establish a dividing line in the concept of sarcopenic obesity in order to better understand this complex pathology.

## 2. Pathogenesis of Visceral and Subcutaneous Fat and Its Relationship with Sarcopenia

Age-related declines in muscle mass and muscle strength may subsequently lead to reduction in physical activity [5]. A decline in muscle mass and physical activity reduces total energy expenditure, which leads to weight gain primarily in the form of visceral abdominal fat [7]. Accumulation of adipose tissue or the presence of adipocyte-infiltrating macrophages leads to increased secretion of proinflammatory cytokines, such as interleukin (IL)-1, IL-6, and tumor necrosis factor-α (TNF-α) [8]. In addition, adipose tissue produces proinflammatory adipokines that promote lipotoxicity in the skeletal muscle cells, thereby contributing to the pathophysiology of sarcopenia [9].

In addition, a routine clinical inflammation biomarker such as C-reactive protein (CRP) has recently been shown to be positively associated with sarcopenia and sarcopenic obesity [10]. CRP, being an acute phase protein, is synthesized by the liver in response to proinflammatory cytokines [11]. Interestingly, Schrager et al. [12] demonstrated in a large sample of elderly individuals that central obesity positively affected inflammation and negatively affected muscle strength, which in turn contributed to the pathogenesis of sarcopenic obesity. Further evidence comes from the recent study of Park et al. [13] who observed that increased high-sensitivity CRP was associated with the presence of sarcopenic obesity in Korean adults. Such findings reinforce the proposed mechanism related to the role of proinflammatory cytokines secreted by metabolically active adipocytes or adipose tissues in the development and progression of sarcopenic obesity [11]. Moreover, during the aging process, the shift of adipose tissue from subcutaneous to visceral adipose sites along with skeletal muscle atrophy leads to an imbalance between proinflammatory adipokines and anti-inflammatory myokines [14]. This unfavorable adipokine/cytokine profile represents a common mechanism for sarcopenia, obesity and immune senescence in elderly populations [15].

It is also hypothesized that infiltration of muscle tissue by adipose tissue is associated with increased inflammation compared to muscle without fat infiltration, thus implying an important link between increased fat mass, triglyceride content in muscle and inflammation [5]. Furthermore, with aging, increased levels of leptin, an adipocyte hormone, may result in leptin resistance and a reduction of fatty acid oxidation in muscles which contributes to ectopic fat deposition in skeletal muscle and other tissues leading to muscle atrophy [16]. Other metabolic abnormalities have been described such as mitochondrial dysfunction, muscle oxidative stress and muscle stem cell dysfunction [17].

Interestingly, there are limited reports on the specific role of subcutaneous peripheral fat accumulation in the pathogenesis of sarcopenic obesity. It has been suggested that increased peripheral fat, specifically leg fat, appears to be associated with a reduction in cardiovascular mortality risk [18]. The protective effect of a large hip circumference may be due to a high concentration of adiponectin, a known anti-inflammatory hormonal protein, and a low rate of lipolysis in the gluteofemoral region [19]. Adiponectin, in particular, is known to inhibit inflammation by blocking nuclear factor κB activation in macrophages and reducing cytokines including TNF-α and IL-6 [20]. Further evidence suggests that subcutaneous fat has a key role in modulating peripheral insulin resistance by regulating visceral fat accumulation [21].

## 3. The Effect of Age on Visceral Fat

Since at least 50% of the increase in visceral fat verifying during the period between 25 and 65 years, strategies for preventing this shift should be focused on this range of age. In addition, based on the cited relationships between visceral adipose tissue and different hormones, the increase in visceral fat with age does not appear to occur until the seventh decade, and decreases greatly between the ages of 60 and 70 years. Moreover, it is suggested that a shift in fat distribution may be prevented to about 60 years of age and even slowed in individuals in their 70s provided weight gain is prevented and activity levels are highly maintained [22].

In another recent review article, Mancuso and Bouchard [23] noted that, in general, the adipose tissue mass increases with age in response to a chronic positive calorie balance, reduced physical activity, and a lower basal metabolic rate. Interestingly, another study by Siervi et al. [24] demonstrated a strong association between age and visceral adiposity in males and females, whereas a significant negative association with subcutaneous adipose tissue was only found in male subjects. As previously mentioned, the mechanism could be explained by a significant negative correlation of basal fat oxidation with fat mass, insulin and triglycerides [25].

As we know, diet and age have a great effect on microbial communities, therefore the associations between age and dietary habits, and the relative abundance of *Blautia* and/or *Bifidobacterium* is still unclear. Another interesting mechanism that could explain the effect of age on visceral adipose tissue was recently demonstrated by Ozato et al. [26] for the first time in the literature that the relative abundance of *Blautia* was not significantly correlated with age, while the relative abundance of *Bifidobacterium* was significantly and inversely correlated with age. However, the data on *Blautia* are contradictory given the fact that *Blautia* was significantly and positively associated with visceral fat mass as estimated by DXA in older adults in another recent study [27].

## 4. Peri-Muscular Fat: A New Entity?

One of the most recent finding that could better describe the effect of obesity on sarcopenia is related to the peri-muscular fat. A recent study by Zhu et al. [28] suggests that Peri-Muscolar fat in older age could further exacerbate the age-related muscular atrophy as examined by the ectopic fat accumulation layered around atrophied hindlimb skeletal muscle. The authors found that the peri-muscular adipose tissue (PMAT) in obese mice attenuated denervation-induced muscle atrophy and suppressed upregulation of genes related to proteolysis and cellular senescence in muscle. In addition, the PMAT accumulation accelerates age- and obesity-induced muscle atrophy by increasing proteolysis and cellular senescence in muscle [28]. Moreover, another study by Morrison [29] showed that PMAT was the strongest determinant of insulin sensitivity/resistance in women with polycystic ovary syndrome. Furthermore, PMAT may interfere with insulin action because it increases local concentrations of free fatty acids or pro-inflammatory cytokines, as well as alterations in insulin diffusion capability, leading ultimately to impairment of insulin action [30].

As defined recently by Kelly et al. [31], fat mass and fat mass index are included to measure obesity, while waist circumference (WC), visceral fat, visceral/subcutaneous fat ratio, intramuscular adipose tissue by BIA and the android to gynoid fat ratio are used to determine the extent of abdominal/visceral fat. In addition, BIA cutoffs were included as it has been previously used to identify osteosarcopenic obesity, a variant phenotype of obesity, mainly observed in older adults [32].

Although the preference is to use DXA, BIA and, whenever possible, computed tomography for magnetic resonance imaging to measure ectopic fat mass and to identify osteosarcopenic obesity, a recent systematic review has suggested that the proxy measures of ectopic fat can be easily used in the field or the clinical settings [31]. Finally, the authors have utilized broadly established cutoffs or those of European origin; however, I further encourage adjustments to the criteria for various ethnic groups.

## 5. Obesity Paradox in Older Adults: Subcutaneous Fat Is the Major Lead

The body mass index (BMI) is the most commonly used measure for classifying overweight and obesity, defined by the World Health Organization (WHO) as a BMI of ≥25, and ≥30 kg/m^2^, respectively [33]. A high BMI is associated with increased mortality from cardiovascular disease (CVD) and certain cancers [34], however the relationship between BMI and all-cause mortality in older age remains uncertain. In their systematic review of elderly adults aged 65 years and above, Janssen and Mark [35] found that BMI in the overweight range was not associated with a significantly increased mortality risk, whereas BMI in the obese range was associated with a moderate increase in mortality risk. Another study that defined sarcopenic obesity using calf skeletal muscle and BMI showed that sarcopenic obesity was not associated with a significantly higher risk of mortality in the community-dwelling elderly population [36]. Similarly, the systematic review and meta-analysis by Flegal et al. [37] showed a significant reduction in all-cause mortality in overweight elderly individuals, although these findings have been questioned since they were related to BMI and not to visceral fat or fat distribution.

Several explanations have been proposed for the paradoxical association between BMI and mortality in older adults. The most probable explanation is that the BMI is not an accurate indicator for adiposity in the elderly as it does not distinguish between body fat mass and body fat-free mass [38]. Further, since BMI measures do not take into account the loss of muscle mass with increasing age, the use of BMI, as a tool for validating cases with obesity-associated co-morbidities, is not as accurate for the elderly population [39].

In the light of the increasing evidence that elderly individuals with several chronic diseases and elevated BMI present a more favorable prognosis compared to individuals who are normal or underweight, a phenomenon known as “the obesity paradox”, it becomes imperative that we explore the positive and negative aspects of being obese at older ages, especially in relation to sarcopenic obesity. In fact, the age-related decline in skeletal muscle function and mass (sarcopenia), is known to have a profound negative impact on health-related quality of life. This means that obesity in older age does not protect from chronic disease-related mortality when it is associated with sarcopenia. The few available studies indeed indicate that low muscle function and mass may be associated with higher mortality in obese individuals with chronic diseases [17].

Nevertheless, the obesity paradox is commonly seen in elderly overweight and obese individuals with coronary heart disease (CHD) or heart failure (HF). In fact, a number of cross-sectional studies have indicated that obese adults have higher cardiovascular risk factors than those with sarcopenic obesity [40,41]. In a meta-analysis involving more than 250,000 patients, Romero-Corral et al. [42] showed that overweight and obese patients with CHD have lower risk of total and CVD mortality compared with underweight and normal weight CHD patients.

## 6. The Effect of Body Composition on Obesity Paradox

The effect of body composition on the obesity paradox is controversial. Shil Hong et al. [43] showed that both BMI and visceral fat area were inversely associated with all-cause mortality over a period of up to 6 years in a sample of Korean elderly individuals. Furthermore, BMI and visceral fat area demonstrated a U-shaped relationship with mortality rates in women. However, total fat mass was not associated with mortality [43]. These findings support those of previous studies indicating an obesity paradox regarding BMI and mortality, basically a higher BMI was narrowly associated with lower mortality [44]. In addition, Perna et al. [4] recently reported that people with the phenotype of subcutaneous sarcopenic obesity had the same risk of frailty and risk factors than sarcopenic elderly but with a lower risk of fractures.

It is well recognized that during periods of starvation, a larger proportion of energy is derived from protein oxidation in the lean compared to the obese state. Moreover, despite the fact that obese individuals have more fat free mass than lean individuals, they tend to lose muscle components (i.e., lean mass) at a slower rate during prolonged starvation compared to their leaner counterparts [45].

The reported benefits to the obese population may be the result of the characteristics of excess adiposity, including extra fat stores, hormones (i.e., insulin, leptin and estrogen) and anti-inflammatory adipokines. Furthermore, the fact that the amount of gained visceral fat decreases with age, in addition to the accumulation of subcutaneous fat stores, may be responsible for the inverse relation between BMI and mortality [46].

## 7. Potential Effects of Hormonal Response and Anti-Inflammatory Adipokines

Body composition is mainly influenced by endocrinological factors, which include hormonal metabolic signals throughout an individual`s lifetime [47]. A frequently observed effect of increased weight gain and expansion of visceral fat is a decrease in blood levels of the adipocyte-derived hormone, adiponectin [48]. Adiponectin depletion leads to reduced efficiency in energy expenditure, reduced glucose utilization, thereby increasing the risk of type 2 diabetes mellitus (T2DM), CVD, colorectal cancer and nonalcoholic fatty liver disease [49,50,51,52].

The aging-related decline in testosterone parallels both the loss in lean body mass and the gain in fat mass which lead to sarcopenic obesity [53]. Levels of this hormone decline by 1% per year and bioavailable testosterone by 2% per year from the age of 30 years in males [54]. Testosterone deficiency further predisposes men to visceral obesity and metabolic syndrome [48]. In fact, moderate obesity was shown to decrease total testosterone mainly as a result of the insulin resistance-associated reduction in sex hormone-binding globulin that binds to testosterone [55]. Further, testosterone supplementation improves the body composition in elderly men by increasing the lean body mass and decreasing the fat mass [56].

Similarly, levels of growth hormone (GH) decline in an age-dependent fashion leading to numerous consequences for skeletal muscle structure and function [47]. The age-related decline in GH results in reduced liver-derived insulin-like growth factor-I (IGF-I), which is one of the main regulators of muscle mass [57]. The combination of GH and testosterone replacement in the sarcopenic elderly population has been reported to have beneficial effects on protein turnover, lipid profile, and fat mass [58].

Aging is also associated with the increased release of glucocorticoids from the adrenal cortex. The stress hormone is known to inhibit protein synthesis and to stimulate proteolysis, producing a catabolic effect in the skeletal muscle [59]. The association between glucocorticoids and excess adiposity is well established clinically and is clearly demonstrated in individuals with Cushing syndrome or patients with sepsis that are characterized by increased visceral adiposity and insulin resistance [60,61].

It has been known for quite some time that physical inactivity leads to visceral fat accumulation in the body, which increases the risk of developing various chronic diseases such as CVD, T2DM, cancer, dementia, and depression. This can be regarded as “the disease of physical inactivity” [62]. Furthermore, the increase in fat mass, especially visceral fat, may lead to chronic inflammation and higher secretion of pro-inflammatory adipokines that further promote insulin resistance, atherosclerosis, neurodegeneration, and tumor growth [12,63]. The health-inducing effect of exercise is regarded as an essential component that helps in promoting the overall health of the body. Evidence shows that the most important organ activated as a result of this physical activity is the skeletal muscle due to greater increases in muscle mass and fiber hypertrophy [64,65].

Research in the past decade revealed an intricate crosstalk between skeletal muscle and the liver and adipose tissue, among other organs, in addition to the recognition of skeletal muscle as an endocrine organ which secretes cytokines called “myokines” [66]. These myokines, which are released from contracting muscle fibers during physical activity have an endocrine hormone-like effect on visceral fat [67]. Other myokines have a more localized effect within the skeletal muscle cells via autocrine or paracrine mechanisms and may be involved in fat oxidation [45]. Some of the myokines that have been found include IL-6, IL-8, IL-15, brain-derived neurotrophic factor (BDNF), leukemia inhibitory factor (LIF), fibroblast growth factor 21, and follistatin-like 1 [68].

Although IL-6 is in principal considered as a pro-inflammatory cytokine produced by macrophages in response to infectious stimuli, IL-6 secretion by skeletal muscle during physical activity, however, exerts anti-inflammatory effects on inflammatory mediators TNF-α and IL-1β, and activation of anti-inflammatory cytokines IL-10 and IL-1ra [69]. This apparent paradox has generated much interest in the metabolic role of IL-6 as it has been shown to have beneficial effects on glucose homeostasis and lipid metabolism [66]. For instance, Petersen et al. [70] demonstrated in vivo that IL-6 infusion enhances systemic fatty acid oxidation and lipolysis in both healthy and T2DM patients.

BDNF, another myokine that is upregulated in skeletal muscle and plasma during exercise has been associated with fat oxidation through the activation of AMP-dependent protein kinase [71]. In addition, BDNF plays a crucial role in learning and memory by regulating survival, growth and maintenance of neurons [72]. The brains of patients with Alzheimer’s disease showed a decline in plasma levels of BDNF concomitant with progression of disease [73]. Thus, the available evidence points to the beneficial role of physical activity in the delicate balance between myokines. Low physical activity worsens both sarcopenic obesity and the accumulation of visceral fat in the body. As visceral fat is increased and skeletal muscles are decreased, the inflammation worsens due to the secretion of pro-inflammatory adipokines that influence lipid metabolism and insulin resistance, creating a vicious cycle that reduces muscle strength and promotes sarcopenia and metabolic syndrome [74].

Inflammation has drawn more attention in recent years due to its strong association with both sarcopenia and obesity [75,76]. For instance, results from the Invecchiare in Chianti (InCHIANTI) study in a sample of community-dwelling individuals older than 65 years showed that sarcopenic obesity defined by WC and grip strength was associated with elevated levels of various inflammatory markers including CRP and IL-6 [12].

In the recent meta-analysis of Bano et al. [77] including over 11,000 adults in 17 studies, sarcopenia was found to be associated with higher serum inflammatory parameters, in particular elevated CRP levels. Similarly, Kim et al. [78] reported that high sensitivity CRP were significant factors predicting sarcopenic obesity in Korean women.

## 8. Clinical Implications of Sarcopenic Obesity

### 8.1. Mortality

There are only a handful of studies exploring the association between sarcopenic obesity and the risk of all-cause of mortality among elderly subjects for different types of phenotypes [79]. For example, Wannamethee et al. [80] reported using WC and mid-arm muscle circumference (MAMC), as a marker for muscle mass, that elderly male subjects with a high WC (>102 cm) and a low MAMC (lowest quartile) had a 55% increase in mortality risk compared with nonsarcopenic, nonobese subjects during the follow-up period of six years. In the follow-up of the same subjects, a 72% increased risk of all-cause mortality, independent of lifestyle and cardiovascular risk factors, was observed in men with sarcopenic obesity [81]. Another prospective study by Batsis et al. [82] showed similar results for sarcopenic obese women with subcutaneous fat based on skeletal muscle mass and body fat measurements using BIA concluding that elderly women with sarcopenia had an increased all-cause mortality risk independent of obesity.

In the New Mexico Aging Process Study, 451 elderly participants aged 60 years and over were followed for 8 years and the prevalence of metabolic syndrome was highest among the nonsarcopenic obese individuals (37.5%) compared to the sarcopenic obese (19.2%) indicating that the metabolic syndrome did not overlap with the researchers’ definition of sarcopenic obesity [83]. Further, the meta-analysis of Sharma et al. [84] who investigated the relationship between BMI and all-cause mortality, CVD mortality, and HF hospitalizations concluded that the risk for CVD mortality and HF hospitalization was lowest in overweight patients while those at the highest risk were in the underweight category.

In another study, a meta-analysis of 9 observational studies with 28,209 patients with chronic HF, Oreopoulos et al. [85] demonstrated that overweight and obese HF patients had reductions in cardiovascular and all-cause mortality compared with HF patients with a normal BMI. Furthermore, the obesity paradox was confirmed by a study on patients after cardiac surgery in which a protective effect of obesity in terms of hospital survival was reported [86]. Protective effects of overweight and obesity have been observed in other chronic diseases such as hypertension [87], stroke [88], atrial fibrillation [89], cancer [90] and type 2 diabetes mellitus (T2DM) [91].

In a cross-sectional study of community-dwelling Taiwanese elderly which assessed sarcopenia using BIA and obesity according to BMI, the findings indicated that sarcopenic obese individuals had the highest risk of metabolic syndrome, with an 11-fold increase in risk compared with the sarcopenic and obese group alone [92]. Moreover, a recent meta-analysis of 12 prospective cohort studies found that sarcopenic obesity is significantly correlated with increased mortality risks in elderly individuals [93]. Similarly, in a cross-sectional study involving 14,299 participants from the National Health and Nutrition Examination Survey III, Srikanthan et al. [94] found that sarcopenic obese individuals had the highest risk of insulin resistance and dysglycemia.

Furthermore, in a recent study by Ji et al. [95], critically ill intra-abdominal septic patients were classified into one of four body composition categories according to the presence or absence of sarcopenia or visceral obesity. The results of the study showed that only sarcopenic obesity was associated with increased risk for 30-day mortality. Therefore, it is important to assess both visceral adipose tissue and muscle areas at the same time in terms of prognostication.

Finally, the recent study by Vann Aller et al. [96] evidenced that BMI did not predict increased or decreased survival time in US adults of 50 years and older. The authors showed for the first time that the DXA based breast cancer phenotype and truncal fat mass/appendicular skeletal muscle mass (ASM) ratio models significantly predicted survival time. However, the association was age-dependent as the participants with sarcopenic obesity were associated with lower survival time in participants between 50 to 70 years, but not in participants older than 70 years.

### 8.2. Cardiovascular Diseases

Inflammatory (CRP, IL-6, etc.) and hemostatic (homocysteine, etc.) markers have been shown to play a crucial role in the development of risk factors associated with CVD in the elderly [97,98].

Cesari et al. [99], however, observed no significant interactions between sarcopenia and obesity with CRP, IL-6 or plasminogen activator inhibitor type 1, the primary inhibitor of the fibrinolytic process, in 286 elderly patients aged ≥55 years with a high CVD risk profile. Nevertheless, Fukuda et al. [100], in a recent study involving 716 individuals, showed that sarcopenic obesity (defined by markers for visceral fat and skeletal muscle index) was significantly associated with incident of CVD even after adjustment for the confounding variables.

### 8.3. Disability and Functional Limitation

Maintaining mobility is an important aspect of health and wellbeing in the elderly population. Several studies have described a positive association between high BMI values and self-reported physical disability or functional limitations among community-dwelling elderly individuals [101,102,103]. Intuitively, having high body fat (along with low lean body mass) may increase the incidence of functional limitation and chronic conditions. Visser et al. [104] reported a similar finding in older obese people, however, low skeletal muscle mass was not assumed to be associated with physical disability or mortality. On the other hand, further evidence has demonstrated an association between low muscle mass and limited physical function [105].

A handful of studies have attempted to evaluate the combined effects of sarcopenia and obesity on physical performance in the elderly. For instance, Baumgarther et al. [83] reported that sarcopenic obese individuals were two to three times more at risk of developing disability associated with reduced activities of daily living than subjects with obesity or sarcopenia alone. In contrast, in a study using data from the National Health and Nutrition Examination Survey study, no significant association was found between sarcopenic obesity and functional limitation among elderly individuals [106]. Another study carried out in Italy with 167 community-dwelling elderly women, aimed at determine the association among muscular strength, functional limitation and physical disability, demonstrated a 3-fold increased risk of functional impairment in the nonsarcopenic obese group, with only a trend towards an increase in risk in the sarcopenic obese group [5].

As clearly explained by Tomlinson et al. [107], the consensus within the literature is that obese individuals have reduced maximum muscle strength relative to body mass in their anti-gravity muscles compared to non-obese persons. This effect on an obese individual is shown to increase the risk of developing osteoarthritis and can potentially cause functional limitations in activities of daily living especially in older adults.

### 8.4. Diabetes

On the other hand, just few studies have looked into the relationship between sarcopenic obesity, metabolic alternations (such as metabolic syndrome and T2DM) and health status. In the cross-sectional study of Messier et al. [40], sarcopenic obese postmenopausal women did not present an unfavorable metabolic profile compared with those who were not sarcopenic obese. Similarly, Baumgartner et al. [83] demonstrated that although T2DM was more frequent in sarcopenic subjects, the prevalence of T2DM was found to be highest in the nonsarcopenic obese group, followed by that of the sarcopenic obese group, and the lowest being in the sarcopenic nonobese group. Conversely, a recent systematic review and meta-analysis of 11 studies showed that sarcopenic obesity increased the risk of T2DM by 38% when compared with sarcopenia or obesity alone [108]. However, these results need to be interpreted with caution as the cross-sectional design of the studies reviewed indicated simple associations between sarcopenic obesity and T2DM without providing definitive information regarding causal relationships between the two conditions.

### 8.5. Liver Cirrhosis

Patients with liver cirrhosis may experience simultaneous loss of skeletal muscle and gain of adipose tissue which lead to sarcopenic obesity [109]. For instance, in the study of Montano-Loza et al. [110], sarcopenia was present in 43% and sarcopenic obesity in 20% of patients. The authors of the same study found that both patients with sarcopenia and with sarcopenic obesity had a high long-term mortality rate than cirrhotic patients without muscular abnormalities. Similarly, Hara et al. [111] explored the prognostic value of skeletal muscle mass and visceral fat accumulation in 161 liver cirrhotic patients. The results of the study showed that sarcopenic patients died more than nonsarcopenic patients, and among patients with sarcopenia, sarcopenic obese patients had the worst prognosis [111].

Furthermore, in a recent report by Eslamparast et al. [112], the authors pointed out that the prevalence of sarcopenic obesity in patients awaiting liver transplantation was estimated to be between 20 to 35%, and an association with increased mortality than observed with either sarcopenia or obesity alone.

### 8.6. Polycystic Ovary Syndrome

Albeit mainly a disorder of excess androgen, polycystic ovary syndrome (PCOS) is frequently associated with insulin resistance, obesity and various metabolic diseases [113]. Women with PCOS, despite their young age, have increased visceral adiposity and more severe insulin resistance as indicated by the homeostatic model assessment of insulin resistance (HOMA-IR) score [114]. Furthermore, visceral adiposity is associated with elevated levels of inflammatory cytokines and serum inflammatory markers including CRP [115].

Nevertheless, the only report in the literature to determine the prevalence of sarcopenic obesity in a sample of women with PCOS is represented by a recent study from McBreairty et al. [115], who showed that 53% of women with PCOS were classified as sarcopenic obese, which is higher than the prevalence in older women reported from the literature.

### 8.7. Cancer

Several systematic reviews and meta-analyses have explored the association of sarcopenic obesity with unfavorable clinical outcomes in patients with different cancers [116,117,118]. For example, Carneiro et al. [119] conducted a meta-analysis including 14 studies linking sarcopenic obesity to clinical outcomes in cancer patients and concluded that sarcopenic obesity was associated with increased surgical complications. Similarly, a recent study by Kobayashi et al. [120] reported that decreased skeletal muscle quality and the accumulation of visceral adipose tissue are closely related to poor patient survival after resection for colorectal liver metastases. Another meta-analysis of 11 studies linking sarcopenic obesity to clinical outcomes in pancreatic cancer patients concluded that the risk of mortality was 1.4 times higher in sarcopenic patients and 2 times higher for sarcopenic obese patients [118]. Lastly, Chen et al. [121] recently demonstrated the impact of sarcopenia and visceral obesity on the surgical management of colorectal cancer, reporting an increased risk of postoperative complications.

### 8.8. Postoperative Implication of Visceral Fat and Sarcopenia

A recent study by Jang et al. [122] was performed to evaluate the predictive values of sarcopenia and visceral obesity measured from preoperative computed tomography/magnetic resonance imaging for postoperative pancreatic fistula (POPF) after pancreaticoduodenectomy in patients with periampullary malignancies. In the univariate logistic regression analysis, both visceral obesity (odds ratio = 2.19) and sarcopenic obesity (odds ratio = 2.65) were predictive of POPF. The results of that study suggest that radiologic quantification of body composition, such as depletion of muscle mass and excessive adipose tissue, might be helpful for predicting POPF in patients following pancreaticoduodenectomy [122].

## 9. Conclusions

Sarcopenic obesity is a complex syndrome that should be better defined in order to manage and guide new therapies and nutritional supplementations. Although there are still no guidelines and criteria for establishing two distinct syndromes between sarcopenic visceral obesity and sarcopenic subcutaneous obesity, this review lays the foundation for defining two new different entities of sarcopenia. This, in turn, will provide new potential therapeutic options for a correct management of this complicated syndrome. Finally, it is strongly recommended that a new diagnostic criterion for the identification of phenotypes be established and that new “obesity-phenotype oriented” clinical trials be conducted in order to provide dedicated personalized individual therapy for sarcopenic obesity.
